# Analysis of the Dirigent Pan-Gene Family in 26 Diverse Inbred Lines Reveals Genomic Diversity in Maize

**DOI:** 10.3390/genes16111285

**Published:** 2025-10-29

**Authors:** Zhihao Liu, Yingjie Xue, Yuxi Xie, Yikun Zhao, Wei Yang, Weiguang Yang, Fengge Wang, Xuejiao Ren

**Affiliations:** 1 College of Agriculture, Jilin Agricultural University, Changchun 130118, China; lzh970206@outlook.com (Z.L.); xieyuxi0824@163.com (Y.X.); davidyoung588@126.com (W.Y.); ywg789@126.com (W.Y.); 2 Maize Research Center, Beijing Academy of Agricultural and Forest Sciences (BAAFS)/Key Laboratory of Crop DNA Fingerprinting Innovation and Utilization (Co-Construction by Ministry and Province), Ministry of Agriculture and Rural Affairs, Shuguang Garden Middle Road No. 9, Beijing 100097, China; yingjiexue_zea@126.com (Y.X.); zhaoqiankaisteam@126.com (Y.Z.)

**Keywords:** maize, dirigent, pan-genome

## Abstract

Background: Dirigent genes play crucial roles in regulating plant architecture development and responses to environmental stress. However, the pan-genomic attributes of these genes remain poorly characterized. Method: The dirigent pan-gene family was reconstructed using the public genome assemblies from the 26 maize Nested Association Mapping project founder lines. Orthogroup classification based on multiple sequence alignment revealed both core and variable family members. Evolutionary pressures were assessed through Ka/Ks ratio analysis, and promoter regions were examined for cis-acting regulatory elements. Haplotype, transcriptomic and genome-wide association study (GWAS) analyses were integrated to explore genetic diversity and functional relevance. Results: Most dirigent members were under purifying selection, whereas a subset may have undergone positive selection. Promoter analysis demonstrated enrichment of stress- and phytohormone-responsive cis-acting regulatory elements, suggesting that regulatory divergence was associated with environmental adaptation. Haplotype analysis revealed allelic diversity among heterotic clusters, potentially contributing to heterosis. Integration with public genome-wide association study datasets identified candidate genes significantly associated with plant architecture and kernel-quality-related traits. Transcriptome profiles indicated that several dirigent genes were preferentially expressed in the roots, suggesting their involvement in root development and nutrient uptake. In addition, public gene expression data showed that certain dirigent genes are induced in response to salt stress, supporting their putative roles in abiotic stress tolerance. Conclusions: These findings provide insights into the molecular mechanisms underlying dirigent gene functions and reveal candidate genes with potential utility for improving maize performance and stress resilience through molecular breeding.

## 1. Introduction

The term “dirigent” is derived from the Latin verb dirigere, meaning to guide, to arrange, or to direct. Dirigent proteins were first isolated from Forsythia intermedia and have since been shown to mediate the regional and stereoselective coupling of coniferyl alcohol-derived free radicals [1]. Dirigent proteins are widely distributed among gymnosperms, angiosperms, and ferns, while dirigent-like proteins are present in non-vascular plants [2]. Dirigent genes are characterized by the presence of a dirigent domain composed of β-folds and loop structures [3]. The encoded proteins typically contain a single dirigent domain, although several family members contain two consecutive dirigent domains, and most lack introns. In addition, in some proteins, a dirigent domain is fused to a domain typical of jacalin-related lectins (JRL) [4].

Dirigent proteins play crucial roles in plant responses to biotic stresses, particularly by contributing to disease resistance through participation in the synthesis of lignin and lignans [5]. Lignin, a widely distributed secondary metabolite in vascular plants, provides mechanical support, facilitates water and substance transport, and enhances resistance to pathogen infection [6]. In the Linum usitatissimum dirigent gene family, certain genes are responsive to 1-naphthaleneacetic acid, abscisic acid, and methyl jasmonate [7].

With the increasing number of plant genome assemblies reported, members of the dirigent gene family have been identified and cloned in a growing number of species, including Arabidopsis thaliana [5], Capsicum annuum [8], rice (Oryza sativa) [9], Gossypium hirsutum [10], and soybean (Glycine max) [11]. However, identifying and analysis of a gene family based on a single reference genome is unlikely to capture the full genetic diversity within an species, thereby limiting our understanding of the structural diversity and evolutionary dynamics of dirigent genes across diverse germplasm. Maize exhibits extensive genomic variation and heterosis among the inbred lines; therefore, a pan-genomic analysis of the dirigent family across multiple maize inbred lines offers a valuable opportunity to uncover both conserved and lineage-specific gene members, as well as regulatory diversification associated with adaptation.

Given advances in genome sequencing technology and the continued reduction in the cost of next- and third-generation sequencing, a growing number of high-quality genomes are being assembled, providing a powerful resource to accelerate the elucidation of gene families’ functional and genomic diversity of gene families [12,13,14,15,16,17]. To date, pan-genomes of various crops, such as soybean [15], rice [18,19], lettuce (Lactuca sativa) [20], and tomato (Solanum lycopersicum) [21], have been constructed. Among these species, a rice super pan-genome, comprising 251 genomes, has been assembled [18]. Pan-genome availability represents a rich data resource that facilitates pan-genomic research on an increasing number of plant species.

Maize is among the most widely planted crops worldwide [22], providing food for humans [23], animal feed, feed, biofuel feedstock, and industrial raw materials [24,25,26]. In addition, maize is an important model plant for the study of gene functions in monocotyledonous plants [27]. In this study, we identified 1224 dirigent genes by analyzing the 26 maize Nested Association Mapping (NAM) project founder lines [28], which were selected to represent the broad genetic diversity of maize. The complete dirigent gene repertoire of maize was defined, and the distribution pattern of cis-acting regulatory elements and the non-synonymous/synonymous substitution rate (Ka/Ks) ratio among orthogroups (OGs) were determined. We identified haplotypes with distinct distribution patterns of dirigent genes across inbred lines belonging to different heterotic clusters. Genome-wide association study (GWAS) results revealed associations between the dirigent genes and multiple important agronomic traits. In addition, RNA expression profiles revealed that certain dirigent genes exhibited root-specific expression and were associated with resistance to abiotic stresses, such as salt stress. The results provide a foundation for the identification and functional analysis of dirigent gene family members in maize, which will expedite exploration of the potential utility of dirigent genes for the genetic improvement of maize.

## 2. Materials and Methods

### 2.1. Identification of Dirigent Genes in the Maize Pan-Genome

The 26 maize genome assemblies comprising the NAM founder lines were downloaded from MaizeGDB (https://www.maizegdb.org (accessed on 2 May 2025)). Scanning of the genome for the Dirigent domain was performed using HMMER 3.4 with an E-value of 1e−5. The hidden Markov model profile of the Dirigent domain was downloaded from InterPro (https://www.ebi.ac.uk/interpro/search/sequence/ (accessed on 1 May 2025)). The structure of the Dirigent domain was confirmed by searching the NCBI Conserved Domain database (CDD; https://www.ncbi.nlm.nih.gov/Structure/cdd/wrpsb.cgi (accessed on 10 May 2025)).

### 2.2. Orthogroup Classification of the Dirigent Pan-Gene Family

To devise an OG classification for Dirigent genes, we used OrthoFinder [29] to conduct the OG classification of dirigent genes; the BLAST 2.17.0 alignment tool used, multiple sequence alignment was used for gene tree inference, and the Markov clustering algorithm parameter was set to 5. We then calculated the paired protein sequence similarity among each single OG group; genes with protein similarity <90% within the same OG group were subgrouped into more homogeneous, smaller groups. Each OG group was required to comprise at least two genes; otherwise, ungrouped genes were treated as singletons.

### 2.3. Analysis of Gene Duplication Events

Segmental duplication events of dirigent genes from the 26 NAM founder lines were identified using MCScanX 1.0.0 with the default parameters [30]. Circos [31] was used to visualize the gene duplication events in each NAM founder line.

### 2.4. Analysis of Cis-Acting Regulatory Elements in Dirigent Genes

The 2 kb sequence upstream of each dirigent gene coding sequence was extracted to conduct an analysis of cis-acting regulatory elements analysis using the PlantCARE online toolkit (https://bioinformatics.psb.ugent.be/webtools/plantcare/html/).

### 2.5. Ka/Ks Calculation

The coding sequence and protein sequence of each dirigent gene in the NAM founder lines were compared using ParaAT 2.0 [32]. The Ka/Ks ratio was calculated with KaKs Calculator 2.0 [33]. Visualization of the Ka/Ks ridgeline plot was performed with the R packages 4.5.1 ggplot2 4.0.0 and ggridges 0.5.7.

### 2.6. Expression Profile Analysis

The RNA sequence data of six different parts for ‘Jing92’ and ‘Jing724’ was polled and sequenced on Illumina platforms with PE150 strategy in Novogene Bioinformatics Technology Co., Ltd. (Beijing, China). The transcriptome data for salt and mannitol treatments was from PRJNA308155. The RNA sequence data were filtered using Trimmomatic [34] with the parameters “SLIDINGWINDOW:4:15 MINLEN:60”. The clean reads were than mapped to the reference genome (B73_V5) using HISAT2 [35]. The read counts for the genes were calculated with featureCounts [36]. Calculation of the fragments per kilobase per million mapped fragments value was performed with R. A heatmap of the expression profile was generated using the R package pheatmap.

### 2.7. GWAS for Dirigent Genes

To evaluate the potential functions of the dirigent genes identified in the maize pan-genome, the GWAS data for agronomic and kernel-quality traits from a public study were obtained [37]. For the agronomic traits, the resample model inclusion probability of 0.05 was chosen as the threshold for associations. The physical position of Dirigent genes based on the B73_V5 reference genome assembly was converted to the position in version 2 with CrossMap [38]. The physical position chain file was obtained from EnsemblPlants (https://plants.ensembl.org/index.html (accessed on 2 June 2025)). Based on the linkage disequilibrium decay in the NAM population, the dirigent genes located within 100 kb of significant association signals were selected as candidate genes.

## 3. Results

### 3.1. Identification of Dirigent Genes in 26 Maize NAM Founder Lines

**Figure 1:**
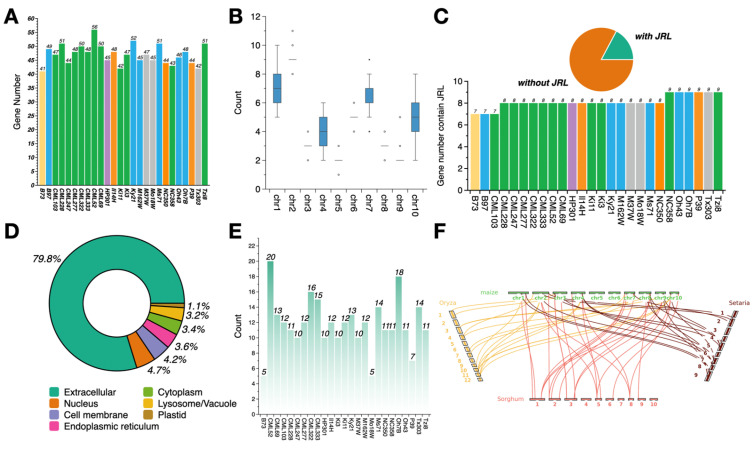
Genomic characterization of the dirigent gene family in maize. (**A**) Distribution of dirigent genes among the 26 NAM founder lines. Different bar colors indicate a different heterotic group: yellow, stiff-stalk; blue, non-stiff-stalk; purple, popcorn; gray, mixed tropical–temperate ancestry; orange, sweet corn; green, tropical. (**B**) Number of dirigent genes on each of the 10 maize chromosomes. (**C**) Number of dirigent genes containing a JRL domain in each founder line. The bar color indicates the same heterotic origin as in (**A**). (**D**) Proportion of dirigent genes localized in different subcellular compartments. (**E**) Number of tandem repeat dirigent genes in 26 NAM founders lines. (**F**) Synteny analysis of dirigent genes between maize and three other graminaceous species (rice, sorghum, and Setaria).

To identify members of the dirigent gene family in the maize pan-genome, we scanned each NAM inbred line genome assembly using the Dirigent domain amino acid sequence as the query (Pfam hidden Markov model: PF03018; E-value threshold, 1 × 10^−5^). Following confirmation of the dirigent domain structure in the NCBI CDD database, we identified 1224 genes containing the dirigent domain across the 26 NAM founder lines [28], with an average of 47 genes per inbred line. The number of dirigent genes in each founder line ranged from 41 (“B73”, stiff-stalk heterotic group) to 56 (“CML52”, tropical group) (Figure 1A). Of the 1224 dirigent genes, 1214 genes were located on chromosomes, whereas the remaining 10 genes were found on scaffolds. Their distribution across the 10 chromosomes was uneven, with the highest number of genes detected on chr2 (235 members) and the fewest on chr5 (57 members) (Figure 1B; Supplementary Table S1).

Based on these results, physicochemical analysis was performed. The molecular weight of the dirigent genes ranged from 7.23 to 78.90 kDa; the majority were concentrated around 20 kDa and a small number were concentrated around 32 kDa. The isoelectric point ranged from 4.12 to 11.66, the instability index ranged from 7.96 to 82.72, and the GRAVY values ranged from −0.59 to 0.55. Less than half of the dirigent genes encoded hydrophilic proteins, whereas the remainder exhibited hydrophobic properties (Supplementary Table S2). Prediction of the subcellular localization (Supplementary Table S5) of the encoded protein (Figure 1D) indicated that almost 80% of the dirigent proteins were extracellular, whereas the remaining genes were localized in compartments like the nucleus and the cell membrane. Each compartment accounted for no more than 5% of the total number.

In addition to the dirigent domain, a subset of the dirigent genes contained a JRL domain, which plays a crucial role in the response to environmental stresses and the regulation of plant development [39]. Of the 1224 dirigent genes, 211 (17.24%) contained the JRL domain (Supplementary Table S3), and each inbred line had 7–9 dirigent genes containing the JRL domain (Figure 1C). Tandem repeat events of dirigent genes in each genome were identified (Figure 1E; Supplementary Table S4), which indicated that the dirigent gene family exhibits significantly divergent tandem repeat patterns (5–20 repeat genes) across the NAM founder lines. Multiple contiguously arranged tandem repeat genes were detected in the genome of certain lines, suggesting that this gene family may have undergone rapid expansion within the genome through tandem duplication. In contrast, other genomes contained only a small number of repeat events, indicating that the dirigent gene family underwent varying degrees of expansion and contraction during the evolutionary history of the inbred lines [40]. In addition, we identified dirigent genes in rice, sorghum, and Setaria. A genome-wide collinearity analysis of dirigent genes in maize and these three graminaceous species (Figure 1F) revealed distinct collinear relationships among dirigent genes across maize and the other three species, which suggests that the genes originated from a common ancestral gene and have been conserved during evolution.

### 3.2. Construction of the Dirigent Pan-Gene Family

To investigate the diversity of the dirigent genes in the maize pan-genome, the 1224 identified dirigent genes in the NAM founder lines were clustered into 68 OGs and 16 singletons (Table S6). Approximately 98.6% of the genes were classified into different OGs and the remaining 1.4% were singletons. Each OG was composed of 2–95 dirigent genes with an average of 18 genes.

**Figure 2:**
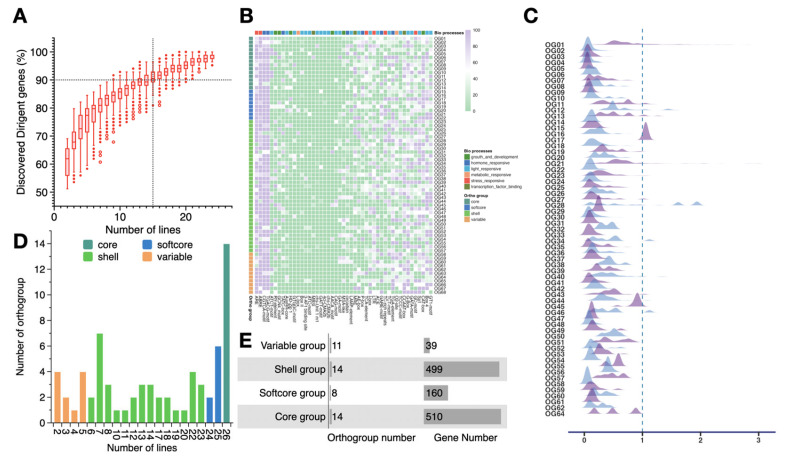
Statistics and properties analysis for the maize dirigent pan-gene family. (**A**) Saturation of dirigent gene family discovery. Boxes indicate fractions of the dirigent pan-gene family recoverable from randomly selected sets of lines of different sizes (1000 bootstrap replications). The vertical dashed line and horizontal dashed line indicate that 90% of dirigent genes can be recovered from 15 inbred lines. (**B**) Distribution of cis-acting regulatory elements in different orthogroups (OGs). The color blocks above the heatmap indicate the categories of cis-acting elements; the color blocks to the left of the heatmap indicate the pan-genome categories of each OG. (**C**) Ka/Ks ratios of different OGs. (**D**) OG size distribution. The bar color indicates different OGs: core, softcore, shell, and variable. (**E**) Genomic composition: number of OGs and genes in the dirigent pan-gene family categories.

We confirmed the saturation of the identified OGs by estimating the OG discovery rate using bootstrap resampling from the 26 NAM founder lines. This revealed that 90% of OGs were detected when 15 inbred lines were randomly selected from among the 68 OGs (Figure 2A). Additional sampling recovered less than 10% additional OGs, which indicates substantial saturation of the maize dirigent pan-genes using the 26 NAM founder lines.

The 2 kb sequence upstream of each dirigent gene was extracted to predict cis-acting regulatory elements. In addition to the core cis-acting elements, we identified several distinct cis-acting elements within the upstream region, such as those for phytohormone response and stress response. Analysis of the cis-acting elements in the promoter region of genes from the various OGs revealed that a high proportion of dirigent genes in most OG groups carry cis-acting elements associated with stress response and phytohormone response, which suggests that these genes may play crucial roles in environmental adaptation and hormone regulation. In contrast, the distribution frequency of cis-acting elements associated with light response was lower in most OGs, indicating that light response may not be a primary regulatory function among dirigent genes (Figure 2B; Table S7). Furthermore, substantial differences were observed among OGs in the proportion of cis-acting elements associated with biological processes, revealing differentiation in the regulatory potential and functional biases of distinct OGs.

**Figure 3:**
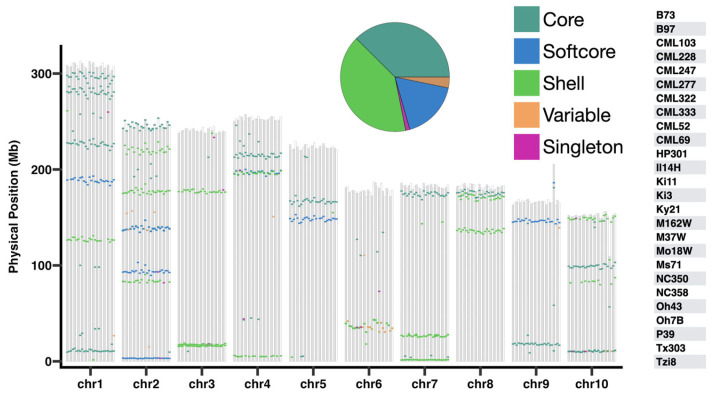
Distribution of dirigent genes among 26 maize NAM founder lines. The physical positions of the dirigent pan-gene family are indicated. The vertical bars represent the 10 chromosomes. The colored boxes indicate dirigent pan-gene categories, the posotion of colored boxes represent the physical position of genes in chromosomes. The pie chart shows the proportion of each pan-gene category.

The ratio of non-synonymous substitutions per non-synonymous site (Ka) to synonymous substitutions per synonymous site (Ks) is considered an indicator of selective pressure acting on the protein [41]. The Ka/Ks ratios of the dirigent genes among the OGs were less than one, indicating that these genes have undergone purifying selection, which suggest that members in the dirigent family are functionally conserved and their protein-coding sequences are under evolutionary constraints. However, certain genes in OG17 and OG28 had Ka/Ks > 1, suggesting that those genes were under positive selection in certain inbred lines, which may contribute to adaptive evolution (Figure 2C). On the basis of the number of NAM founder lines in the OGs, we classified each OG into a core set (present in all 26 inbred lines), a softcore set (present in 24 or 25 inbred lines), a shell set (present in 6–23 inbred lines), and a variable set (present in 5 or less inbred lines) (Figure 2D, Figure 3). More than 40% of the genes were present in all 26 founder lines, whereas 3.18% of the genes were detected in 5 or fewer founder lines.

### 3.3. Maize Germplasm Haplotypes of the Dirigent Pan-Gene Family

**Figure 4:**
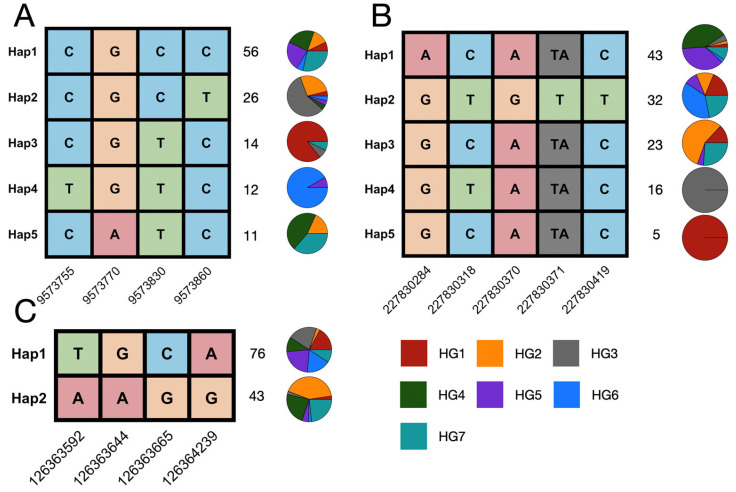
Haplotypes of dirigent genes and their distribution among seven heterotic clusters. (**A**–**C**) The haplotypes for Zm00001eb003590, Zm00001eb043230, and Zm00001eb026860. Each column represents a polymorphic site, and each row represents a distinct haplotype. The nucleotide variants at each site are color-coded, and the positions of the SNPs are indicated at the bottom. The number on the right side of each haplotype indicates the total number of individuals carrying that haplotype. The pie charts illustrate the proportional distribution of each haplotype across seven heterotic clusters (HG1-HG7), as indicated by the color key at the bottom.

To evaluate the genomic variation in dirigent genes in modern maize inbred lines, we conducted variation calling for the dirigent pan-gene family using second-generation sequencing data for 150 maize inbred lines generated in a previous study [42]. The inbred lines comprised seven heterotic clusters (HG1 to HG7). A haplotype analysis based on this variation revealed a highly heterogeneous haplotype distribution pattern within the dirigent pan-gene family. Certain dirigent genes, represented by Zm00001eb003590 (Figure 4A), had a high proportion of the primary haplotype (Hap 1) in most clusters, whereas the secondary haplotypes (Hap 2 to Hap 4) were enriched in a specific cluster (HG3, HG1, and HG6), which suggests that this haplotype may be associated with the local adaptation of the cluster or a rare phenotype. In contrast, a different proportion of the dirigent genes, such as Zm00001eb026860 (Figure 4C), had a polycentric haplotype structure, with several haplotypes composed of multiple clusters in different proportions. It is particularly noteworthy that for certain genes, such as Zm00001eb043230 (Figure 4B), the main haplotype had a high proportion of HG4 and HG5, with very low proportions of other heterotic clusters. Some clusters (HG1, HG2, and HG3) formed a distinct, rare haplotype. This relatively skewed haplotype distribution pattern suggests that dirigent genes may have experienced strong positive selection in the ancestors of HG4 and HG5, leading to the rapid fixation of haplotypes.

### 3.4. Association of Dirigent Genes with Agronomic Traits and Their Expression Profiles

**Figure 5:**
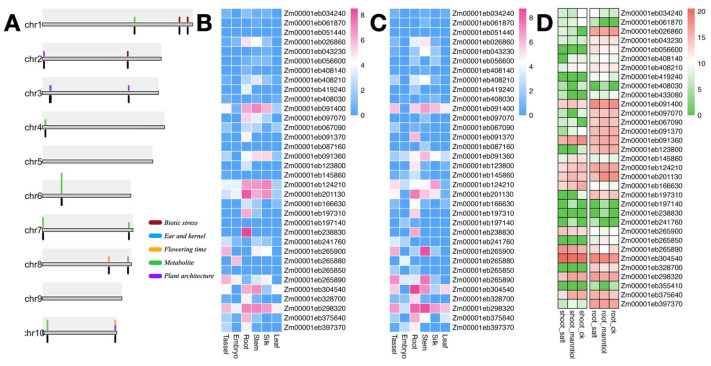
GWAS candidate genes and expression profiles of dirigent genes. (**A**) Distribution of dirigent genes and GWAS candidate loci on the 10 maize chromosomes. Colored blocks above the chromosome represent candidate loci, and black blocks below the chromosome represent the dirigent genes associated with the trait. (**B**,**C**) Expression profiles of dirigent genes in the leaf, silk, stem, embryo, and tassel of ‘Jing92’ and ‘Jing724’. (**D**) Expression profiles of dirigent genes in the shoot and root under exposure to salt and mannitol stresses.

To investigate the functions of dirigent genes in maize, the GWAS data for agronomic traits from a previous study [43] were obtained. On the basis of the linkage disequilibrium decay of the GWAS single-nucleotide polymorphism markers in the NAM population, we selected the regions within ±100 kb of GWAS association signals as candidate gene regions based on the physical position. The association signals for 21 dirigent genes with agronomic traits were determined (Figure 5A; Table S8). These genes were primarily associated with plant architecture and stress response, as well as certain kernel-quality-related traits. In addition, some dirigent genes were associated with metabolite synthesis of which 15 dirigent genes were significantly associated with a single trait and others were significantly associated with two or more traits.

To investigate the expression patterns of the dirigent pan-gene family in different plant tissues, we selected “Jing92”and “Jing724” (a parental line of “Jingke 968”) and performed RNA sequencing on six tissue types: embryo, leaf, root, tassel, stem, and silk. Gene expression levels were calculated through standardized processing. Expression analysis revealed highly consistent tissue-specific expression patterns for the dirigent genes in “Jing92”and “Jing724” (Figure 5B,C), indicating robust stability of the dirigent gene expression profile across different genetic backgrounds. Specifically, dirigent genes exhibited generally low expression levels in the embryo and leaf and high expression levels in the root and stem, with the strongest expression signals observed in the root. These results suggest a potential role for dirigent genes in root biological processes. Based on previous findings [34], members of the dirigent gene family are known to participate in the plant lignin biosynthesis pathway and the regulation of Casparian strip deposition. Therefore, we speculate that their expression patterns are strongly associated with lignin accumulation and Casparian strip formation in the roots, which may provide an important functional context for the high expression levels of this gene family in root tissues.

To further investigate the expression patterns of dirigent genes exposed to various abiotic stress, we obtained transcriptome data for abiotic and non-stress treatments from public databases (PRJNA308155). The stress treatments included salt and mannitol treatments. The expression profiles under salt stress and mannitol stress in roots and leaves, respectively, indicated that dirigent genes involved in stress responses exhibited distinct expression patterns between the two tissues; compared with those in the shoot, a greater number of dirigent genes showed higher basal expression levels in the root. In response to the salt and mannitol stress treatments, the expression levels of certain dirigent genes changed. In the root, certain genes showed no significant change in expression level before and after exposure to salt stress, whereas other genes exhibited specific upregulation in response to salt stress. This expression pattern suggests that such genes may play crucial roles in root-specific stress responses, indicating that salt stress-related signaling pathways may exhibit higher constitutive activity or induction sensitivity in roots. Furthermore, the insensitivity of the expression levels of some dirigent genes to stress in the roots may reflect their stress-independent physiological functions within root tissues (Figure 5D).

## 4. Discussion

The dirigent domain is among the most abundant and interactive domains in eukaryotic genomes. Dirigent genes play an important role in plant secondary metabolism and defense responses [43]. Compared with previous dirigent gene family studies in other crops such as rice and soybean, our findings highlight the distinctive evolutionary and functional characteristics of dirigent members in maize. We reconstructed the dirigent pan-gene family across 26 maize NAM founder lines, which provided an evolutionary framework for exploring gene divergence. Multiple levels of genomic diversity have not been observed in other crops such as soybean or rice. The Ka/Ks ratios among the dirigent OGs indicated that genes with a high ratio have undergone purifying selection, suggesting that members of the dirigent family are functionally conserved and that their protein-coding sequences are under evolutionary constraints to maintain essential structural or catalytic roles. However, a subset of genes showed signatures for positive selection, implying that the genes have possible adaptive roles under specific ecological contexts. These signals of adaptive evolution could reflect functional diversification or lineage-specific adaption, possibly related to environmental stress tolerance or variations in developmental processes. These findings indicate that while the dirigent gene family as a whole is evolutionarily conserved, some members have undergone sequence diversification that may contribute to the functional and adaptive differentiation within maize. Consistent with this suggestion, the analysis of cis-acting regulatory elements revealed the enrichment of stress response and phytohormone response elements in the majority of dirigent OGs, whereas light response motifs were less frequently represented. This finding suggests that the dirigent gene family is primarily engaged in stress-related and developmental pathways, with diversified promoter structures across different OGs. The presence of multiple hormone-responsive elements, such as TGA and CGTCA motifs, indicates that dirigent genes may participate in auxin- and jasmonic acid-mediated signaling networks that coordinate growth adaptation.Moreover, the abundance of stress-responsive elements implies that these members may be transcriptionally activated under abiotic stress such as drought or salinity. The variation in cis-acting elements composition across different OGs reflects promoter-level diversification, which could lead to distinct spatial or temporal expression patterns and facilitate functional specialization within the dirigent gene family.

The diversification of the dirigent family may have accompanied its domestication and subsequent adaptation to diverse agroecological environments. The expansion and functional divergence of certain dirigent orthogroups, together with the enrichment of hormone- and stress-responsive cis-acting elements, suggest that these genes might have contributed to the enhancement of stress resilience and structural reinforcement during maize’s improvement.

Haplotype analysis among the seven distinct heterotic clusters further demonstrated allelic variation among dirigent gene family members, which may underlie functional divergence in hybrid performance. Furthermore, in combination with GWAS results based on previous datasets, a proportion of dirigent family members were significantly associated with several important agronomic traits, such as plant architecture, biotic stress, and flowering time. Dirigent proteins play a critical role in important cellular and organismal metabolic processes, and participate in plant development and environmental interactions. However, the functions of the majority of dirigent genes remain uncertain. Transcriptome profiles revealed distinct expression patterns of dirigent gene family members, with some of the genes showing preferential expression in the root. These findings suggest that the roots harbor a more complex and differentiated transcriptional regulatory network, potentially exhibiting greater functional specificity and adaptability in response to osmotic stress. This suggestion is consistent with the findings of a previous study [43], which found that dirigent genes promote salt stress adaptation by regulating the deposition of the Casparian strip. Analysis of predicted cis-acting regulatory elements further supports the potential role of the dirigent gene family in abiotic stress responses.

The results of this study provide valuable insights and genetic resources for maize molecular breeding. The identification of *dirigent* genes under positive selection and those enriched with stress- and hormone-responsive cis-acting elements highlights their potential involvement in adaptive evolution and environmental resilience. These genes, particularly those showing haplotype differentiation among heterotic groups, could be exploited as candidate loci for improving stress tolerance, plant architecture, and yield-related traits. Integrating these candidate genes into breeding programs through marker-assisted selection or genome editing may facilitate the development of maize varieties with enhanced adaptability and performance under variable environmental conditions.

## 5. Conclusions

In this study, dirigent gene family members of the 26 NAM founder lines were analyzed, resulting in the identification of 1224 maize dirigent genes. The results provide a pan-genomic overview of the maize dirigent gene family, revealing both evolutionary conservation and functional diversification. The coexistence of core and variable members highlights the balance between maintaining essential functions and enabling adaptation. The Ka/Ks ratios and predicted cis-acting regulatory elements revealed divergent evolutionary pressures and regulatory patterns. Haplotype and GWAS analyses identified candidate genes associated with important agronomic traits. Transcriptome and public expression data further suggested that dirigent genes play important roles in root development and salt stress responses. Collectively, these findings demonstrate that the maize dirigent gene family represents a dynamic gene suite that participates in developmental processes and environmental adaptation, and contains promising targets for molecular breeding and crop improvement.

## Data Availability

The original contributions presented in this study are included in the article/Supplementary Material. Further inquiries can be directed to the corresponding author.

## Supplementary Materials

The following supporting information can be downloaded at: https://www.mdpi.com/article/10.3390/genes16111285/s1, Table S1: The number of dirigent genes identified in 26 NAM founders; Table S2: The physicochemical properties of dirigent genes; Table S3: Dirigent members containing JRL domain; Table S4: Tandem repeat event gene pair of dirigent genes; Table S5: Prediction of the subcellular localization of dirigent members; Table S6: Dirigent family genes with corresponding inbred lines and OrthoGroup classifications; Table S7: Analysis of cis-acting elements in the promoter regions of the dirigent family members; Table S8: Summary of associations between dirigent genes and agronomic traits.
